# Supplementary material for: CRISPRi is not strand-specific at all loci and redefines the transcriptional landscape
Source: eLife. 2017 Oct 23;6:e29878. doi: 10.7554/eLife.29878 (PMC5665645; doi:10.7554/eLife.29878)
Supplement: Supplementary file 1. — (A) Templates for sgRNAs (B) Primers for strain construction (C) Saccharomyces cerevisiae strains used in this study (D) Primers for construction of Northern blot probe templates (E) Primers for real-time qPCR at HMS2 (F) Primers for 3’ RACE mapping of the GAL1 sense transcript. [file elife-29878-supp1.docx]

**Supplementary File 1A. Templates for sgRNAs**

| **sgRNA template** | **Sequence 5’-3’** |
| --- | --- |
| Constant region | AGACATAAAAAACAAAAAAAGCACCACCGACTCGGTGCCACTTTTTCAAGTTGATAACGGACTAGCCTTATTTTAACTTGCTATTTCTAGCTC |
| *HMS2* S+77NT variable region | TAAAACGATGTCTTTGTAAGCAAACT |
| *HMS2* S+106NT variable region | TAAAACGCTGCAGGGGAACGCTTACT |
| *HMS2* S+179NT variable region | TAAAACGACCAGTTCACCAAAGTCAT |
| *HMS2* AS+243NT variable region | TAAAACCGGGATGCAACTGCGGGTGC |
| *HMS2* AS+276NT variable region | TAAAACTATCCAAAGAGCAGTTCATA |
| *HMS2* AS+59NT variable region | TAAAACACTGCAAAAAATGGTGGAAA |
| *HMS2* S+77NT  full-length | AGACATAAAAAACAAAAAAAGCACCACCGACTCGGTGCCACTTTTTCAAGTTGATAACGGACTAGCCTTATTTTAACTTGCTATTTCTAGCTCTAAAACGATGTCTTTGTAAGCAAACT |
| *HMS2* S+106NT  full-length | AGACATAAAAAACAAAAAAAGCACCACCGACTCGGTGCCACTTTTTCAAGTTGATAACGGACTAGCCTTATTTTAACTTGCTATTTCTAGCTCTAAAACGCTGCAGGGGAACGCTTACT |
| *HMS2* S+179NT  full-length | AGACATAAAAAACAAAAAAAGCACCACCGACTCGGTGCCACTTTTTCAAGTTGATAACGGACTAGCCTTATTTTAACTTGCTATTTCTAGCTCTAAAACGACCAGTTCACCAAAGTCAT |
| *HMS2* AS+243NT  full-length | AGACATAAAAAACAAAAAAAGCACCACCGACTCGGTGCCACTTTTTCAAGTTGATAACGGACTAGCCTTATTTTAACTTGCTATTTCTAGCTCTAAAACCGGGATGCAACTGCGGGTGC |
| *HMS2* AS+276NT  full-length | AGACATAAAAAACAAAAAAAGCACCACCGACTCGGTGCCACTTTTTCAAGTTGATAACGGACTAGCCTTATTTTAACTTGCTATTTCTAGCTCTAAAACTATCCAAAGAGCAGTTCATA |
| *HMS2* AS+59NT  full-length | AGACATAAAAAACAAAAAAAGCACCACCGACTCGGTGCCACTTTTTCAAGTTGATAACGGACTAGCCTTATTTTAACTTGCTATTTCTAGCTCTAAAACACTGCAAAAAATGGTGGAAA |
| *GAL1* AS+28NT  full-length | CAAATTTTAAAGTGACTCTTGTTTTAGAGCTAGAAATAGCAAGTTAAAATAAGGCTAGTCCGTTATCAACTTGAAAAAGTGGCACCGAGTCGGTGGTGCTTTTTTTGTTTTTTATGTCT |
| *GAL1* AS+112NT  full-length | TGAGTTCAAACCGCAGTTGAGTTTTAGAGCTAGAAATAGCAAGTTAAAATAAGGCTAGTCCGTTATCAACTTGAAAAAGTGGCACCGAGTCGGTGGTGCTTTTTTTGTTTTTTATGTCT |
| *GAL1* AS+93T  full-length | TTTTCATGGTTTTTTAATTGGTTTTAGAGCTAGAAATAGCAAGTTAAAATAAGGCTAGTCCGTTATCAACTTGAAAAAGTGGCACCGAGTCGGTGGTGCTTTTTTTGTTTTTTATGTCT |

**Supplementary File 1B. Primers for strain construction**

| **Primer** | **Sequence 5’-3’** |
| --- | --- |
| *URA3* insertion into *SNR52* F (template = pRS316) | GATGAATGACATTAGCGTGAACAATCTCTGATACAAAATCCTGAGAGTGCACCACGCTTT |
| *URA3* insertion into *SNR52* R (template = pRS316) | CAGAAGGAAGGCAACATAAGTTTTTCTAATCCTAAAATCTGTGCGGTATTTCACACCGCA |
| sgRNA *HMS2* S+77NT insertion into *SNR52* F | TCGGCGTTCGAAACTTCTCCGCAGTGAAAGATAAATGATCAGTTTGCTTACAAAGACATC |
| sgRNA *HMS2* S+106NT insertion into *SNR52* F | TCGGCGTTCGAAACTTCTCCGCAGTGAAAGATAAATGATCAGTAAGCGTTCCCCTGCAGC |
| sgRNA *HMS2* S+179NT insertion into *SNR52* F | TCGGCGTTCGAAACTTCTCCGCAGTGAAAGATAAATGATCATGACTTTGGTGAACTGGTC |
| sgRNA *HMS2* AS+243NT insertion into *SNR52* F | TCGGCGTTCGAAACTTCTCCGCAGTGAAAGATAAATGATCGCACCCGCAGTTGCATCCCG |
| sgRNA *HMS2* AS+276NT insertion into *SNR52* F | TCGGCGTTCGAAACTTCTCCGCAGTGAAAGATAAATGATCTATGAACTGCTCTTTGGATA |
| sgRNA *HMS2* AS+59NT insertion into *SNR52* F | TCGGCGTTCGAAACTTCTCCGCAGTGAAAGATAAATGATCTTTCCACCATTTTTTGCAGT |
| sgRNA *GAL1* AS+28NT insertion into *SNR52* F | TCGGCGTTCGAAACTTCTCCGCAGTGAAAGATAAATGATCCAAATTTT AAAGTGACTCTTG |
| sgRNA *GAL1* AS+112NT insertion into *SNR52* F | TCGGCGTTCGAAACTTCTCCGCAGTGAAAGATAAATGATCTGAGTTCAAACCGCAGTTGA |
| sgRNA *GAL1* AS+93T insertion into *SNR52* F | TCGGCGTTCGAAACTTCTCCGCAGTGAAAGATAAATGATCTTTTCATG GTTTTTTAATTGG |
| sgRNA insertion into *SNR52* R | AGAGGTGGCATTTTACATAACAATAGTGACAAAAAATAAAAGACATAAAAAACAAAAAAA |
| *SNR52* check F | AGCAAACAGCCACTGACTGT |
| *SNR52* check R | GCGTACTAATGTTTTTTGTT |
| *SNR52* Sequencing F | TTGTAGTGCCCTCTTGGGCTAG |

**Supplementary File 1C. Yeast strains used in this study**

| **Strain name** | **Parent** | **Genotype** | **Source** |
| --- | --- | --- | --- |
| BY4741 | S288C | MATa; *his3Δ 1; leu2Δ 0; met15Δ 0; ura3Δ* | Euroscarf |
| snR52::URA3 + dCas9 | BY4741 | snR52::*URA3* + p*TDH3*-dCas9 (Addgene plasmid 46920, *LEU*) | This study |
| *HMS2* S+77NT | snR52::URA3 +dCas9 | snR52::sgRNA *HMS2* S+77NT | This study |
| *HMS2* S+106NT | snR52::URA3 +dCas9 | snR52::sgRNA *HMS2* S+106NT | This study |
| *HMS2* S+179NT | snR52::URA3 +dCas9 | snR52::sgRNA *HMS2* S+179NT | This study |
| *HMS2* AS+243NT | snR52::URA3 +dCas9 | snR52::sgRNA *HMS2* AS+243NT | This study |
| *HMS2* AS+276NT | snR52::URA3 +dCas9 | snR52::sgRNA *HMS2* AS+276NT | This study |
| *HMS2* AS+59NT | snR52::URA3 +dCas9 | snR52::sgRNA *HMS2* AS+59NT | This study |
| *HMS2* S+77NT *xrn1* | *HMS2* S+77NT | *xrn1*::HISMX6 | This study |
| *HMS2* S+106NT *xrn1* | *HMS2* S+106NT | *xrn1*::HISMX6 | This study |
| *HMS2* S+179NT *xrn1* | *HMS2* S+179NT | *xrn1*::HISMX6 | This study |
| *HMS2* AS+243NT *xrn1* | *HMS2* AS+243NT | *xrn1*::HISMX6 | This study |
| *HMS2* AS+276NT *xrn1* | *HMS2* AS+276NT | *xrn1*::HISMX6 | This study |
| *HMS2* AS+59NT *xrn1* | *HMS2* AS+59NT | *xrn1*::HISMX6 | This study |
| BY4741 pRS315 | BY4741 | +pRS315 | This study |
| *HMS2:URA3* | BY4741 | *HMS2 Δ(1-1076)::URA3* | (Nguyen et al. 2014) |
| *HMS2:URA3* pRS315 | *HMS2:URA3* | +pRS315 | This study |
| *GAL1::ADH1*t | BY4741 | *GAL1 Δ(758-1587)::ADH1*t | (Murray et al. 2012) |
| TATA mutant | BY4741 | *GAL1 Δ(758-1587)::ADH1*t (scramble TATA #1) | (Murray et al. 2015) |
| *GAL1::ADH1*t *xrn1* | *GAL1::ADH1*t | *xrn1::KANMX6* | This study |
| TATA mutant *xrn1* | TATA mutant | *xrn1::HISMX6* | This study |
| *GAL1::ADH1*t snR52::URA3 +dCas9 | *GAL1::ADH1*t | snR52::*URA3* + p*TDH3*-dCas9 (Addgene plasmid 46920, *LEU*) | This study |
| *GAL1* AS+28NT | *GAL1::ADH1*t snR52::URA3 | snR52::sgRNA *GAL1* AS+28NT | This study |
| *GAL1* AS+112NT | *GAL1::ADH1*t snR52::URA3 + dCas9 | snR52::sgRNA *GAL1* AS+112NT | This study |
| *GAL1* AS+93T | *GAL1::ADH1*t snR52::URA3 +dCas9 | snR52::sgRNA *GAL1* AS+93T | This study |
| *GAL1* AS+112NT *xrn1* | *GAL1* AS+112NT | *xrn1::KANMX6* | This study |

**Supplementary File 1D. Primers for construction of Northern probe templates**

| **Primer** | **Sequence 5’-3’** |
| --- | --- |
| *HMS2* H1 S F | GACCCGTCCTTCTTTTACCC |
| *HMS2* H1 S T7 R | TAATACGACTCACTATAGGGAGAGACACACTTGGCGACTG |
| *HMS2* H1 AS T7 F | TAATACGACTCACTATAGGGGACCCGTCCTTCTTTTACCC |
| *HMS2* H1 AS R | AGAGACACACTTGGCGACTG |
| *HMS2* H2 S F | GGCGGGCCGCCCGGACTGCG |
| *HMS2* H2 S T7 R | TAATACGACTCACTATAGGGCGGTTTGGTAGGCGTGAAAC |
| *HMS2* H2 AS T7 F | TAATACGACTCACTATAGGGGGCGGGCCGCCCGGACTGCG |
| *HMS2* H2 AS R | CGGGTGCCACTGAGTGGCCG |
| *HMS2* H3 S F | GTTTCA CGCCTACCAA ACCG |
| *HMS2* H3 S T7 R | TAATACGACTCACTATAGGGCGGGTGCCACTGAGTGGCCG |
| *HMS2* H4 S F | TACGACCTTC TTTTCGTCAC |
| *HMS2* H4 S T7 R | TAATACGACTCACTATAGGGCGGGTGCCACTGAGTGGCCG |
| *HMS2* H4 AS T7 F | TAATACGACTCACTATAGGGTACGACCTTC TTTTCGTCAC |
| *HMS2* H4 AS R | GAATGACCCTCAGAGGCAGG |
| *BAT2* S F | AGGCTTACAGAACGGTGGAC |
| *BAT2* S T7 R | TAATACGACTCACTATAGGGGCTCTATCAGGCGTGGAAAC |
| *GAL1* F | TGTGTCGGACTGGTCTAATT |
| *GAL1* R | GATCCATACCGCCATTGTTA |
| 18S F | AGTGAAACTGCGAATGGCTC |
| 18S R | TGCTGGCACCAGACTTGCCC |

**Supplementary File 1E. Primers for real-time qPCR at *HMS2***

| **Primer** | **Sequence 5’-3’** |
| --- | --- |
| *HMS2* +669 (A) F | ACGCTACTCGTTGCTGTTAC |
| *HMS2* +835 (A) R | ACACGGTGACGAAAAGAAGG |
| *HMS2* +870 (B) F | CAAGAGACTGAGAAACCTAC |
| *HMS2* +1044 (B) R | AAGTTGTGTTGGGGTGAAGG |

**Supplementary File 1F. Primers for 3’ RACE mapping of the *GAL1* sense transcript**

| **Primer** | **Sequence 5’-3’** |
| --- | --- |
| Adapter Primer (AP) | GCCCACGCGTCGACTAGTACTTTTTTTTTTTTTTTT |
| Universal amplification primer (UAP) | GCCCACGCGTCGACTAGTAC |
| *GAL1* +346 F | TTGATCCTTCTGTGTCGGACTG |
