# Supplementary material for: CRISPRi is not strand-specific at all loci and redefines the transcriptional landscape
Source: eLife. 2017 Oct 23;6:e29878. doi: 10.7554/eLife.29878 (PMC5665645; doi:10.7554/eLife.29878)
Supplement: Transparent reporting form [file elife-29878-transrepform.docx]

***eLife’s* transparent reporting form**

We encourage authors to provide detailed information *within their submission* to facilitate the interpretation and replication of experiments. If you have any questions, please contact us: [editorial@elifesciences.org](mailto:editorial@elifesciences.org).

**Sample-size estimation**

- You should state whether an appropriate sample size was computed when the study was being designed
- You should state the statistical method of sample size computation and any required assumptions
- If no explicit power analysis was used, you should describe how you decided what sample (replicate) size (number) to use

Please outline where this information can be found within the submission (e.g., page numbers or figure legends), or explain why this information doesn’t apply to your submission:

We did not perform a power analysis as the vast majority of the data in this manuscript is from Northern blotting. To raise the stringency of our results, we performed at least 4 independent biological replicates to support the central findings of the paper.

**Replicates**

- You should report how often each experiment was performed
- You should include a definition of biological versus technical replication
- The data obtained should be provided and sufficient information should be provided to indicate the number of independent biological and/or technical replicates
- If you encountered any outliers, you should describe how these were handled
- Criteria for exclusion/inclusion of data should be clearly stated
- High-throughput sequence data should be uploaded before submission, with a private link for reviewers provided (these are available from both GEO and ArrayExpress)

Please outline where this information can be found within the submission (e.g., page numbers or figure legends), or explain why this information doesn’t apply to your submission:

Details about the number of replicates is found in the figure legends. All replicates are independent biological repeats. No outliers were removed.

(The manuscript does not contain or reference any high-throughput sequencing data.)

**Statistical reporting**

- Statistical analysis methods should be described and justified
- Raw data should be presented in figures whenever informative to do so (typically when N per group is less than 10)
- For each experiment, you should identify the statistical tests used, exact values of N, definitions of center, methods of multiple test correction, and dispersion and precision measures (e.g., mean, median, SD, SEM, confidence intervals; and, for the major substantive results, a measure of effect size (e.g., Pearson's r, Cohen's d)
- Report exact p-values wherever possible alongside the summary statistics and 95% confidence intervals. These should be reported for all key questions and not only when the p-value is less than 0.05.

All statistical tests performed have been T-tests. The number of replicates for each experiment is detailed in the Figure Legends. Exact p values have been stated in the text where appropriate. The error bars on all bar charts show the SEM.

Please outline where this information can be found within the submission (e.g., page numbers or figure legends), or explain why this information doesn’t apply to your submission:

(For large datasets, or papers with a very large number of statistical tests, you may upload a single table file with tests, Ns, etc., with reference to page numbers in the manuscript.)

**Additional data files (“source data”)**

- We encourage you to upload relevant additional data files, such as numerical data that are represented as a graph in a figure, or as a summary table
- Where provided, these should be in the most useful format, and they can be uploaded as “Source data” files linked to a main figure or table
- Include model definition files including the full list of parameters used
- Include code used for data analysis (e.g., R, MatLab)
- Avoid stating that data files are “available upon request”

Please indicate the figures or tables for which source data files have been provided:

Raw data (uncropped scans of Northern and Western blots, raw Sanger sequencing data, qPCR run files) for all figures will be uploaded onto Mendeley data upon acceptance. A Source Data File (in Excel format) has been uploaded with the submission containing all the quantified Northern, yeast growth curve and qPCR efficiency signals and the analysis to produce the graphs in the figures. This Source Data File has been referenced where appropriate in the Figure legends. All the Supplementary Tables referenced in the Materials and Methods have been combined into Supplementary File 1 and referenced accordingly.
